# Finite element study on modification of bracket base and its effects on bond strength

**DOI:** 10.1590/2176-9451.20.2.076-082.oar

**Published:** 2015

**Authors:** Tarulatha R. Shyagali, Deepak P. Bhayya, Chandralekha B. Urs, Shashikala Subramaniam

**Affiliations:** 1 Professor, Darshan Dental College and Hospital, Department of Orthodontics and Dentofacial Orthopedics, Udaipur, India; 2 Professor, Darshan Dental College and Hospital, Loyara, Department of Pediatric and Preventive Dentistry, Udaipur, India; 3 Former professor and head, Vaidehi Dental College and Hospital, Department of Orthodontics and Dentofacial Orthopedics, Bangalore, India; 4 Professor, KGM Dental College and Hospital, Department of Orthodontics and Dentofacial Orthopedics, Kolar, India

**Keywords:** Finite element analysis, Orthodontic brackets, Mechanical stress

## Abstract

**OBJECTIVE:**

This article aims to analyze the difference in stresses generated in the bracket-cement-tooth system by means of a peel load in single and double-mesh bracket bases using a three-dimensional finite element computer model.

**MATERIAL AND METHODS:**

A three-dimensional finite element model of the bracket-cement-tooth system was constructed and consisted of 40,536 bonds and 49,201 finite elements using a commercial mesh generating programmer (ANSYS 7.0). Both single and double-mesh bracket bases were modified by varying the diameter from 100-400 µm progressively, and the spacing between the mesh wires was kept at 300 µm for each diameter of wire. A peel load was applied on the model to study the stresses generated in different layers.

**RESULTS:**

In case of double-mesh bracket base, there was reduction in stress generation at the enamel in comparison to single-mesh bracket base. There was no difference in stress generated at the bracket layer between single and double-mesh bracket bases. At the impregnated wire mesh (IWM), layer stresses increased as the wire diameter of the mesh increased.

**CONCLUSION:**

Results show that bracket design modification can improve bonding abilities and simultaneously reduce enamel damage while debonding. These facts may be used in bringing about the new innovative bracket designs for clinical use.

## Abstract (pt)

**OBJETIVO:**

o objetivo do presente artigo é analisar a diferença entre as tensões geradas na interface braquete-cemento-dente por meio do teste *peel load* em bases de braquete de malha simples e dupla e do método de elementos finitos tridimensional.

**MÉTODOS:**

foi construído um modelo de elementos finitos do sistema composto pela interface braquete-cemento-dente. Esse modelo consistiu de 40.536 nós e 49.201 elementos finitos. A análise foi feita com a ajuda do programa ANSYS 7.0. Tanto a base de braquete de malha única quanto a de malha dupla sofreram modificações no diâmetro, que variou de 100 a 400µm, progressivamente. O espaço entre os fios das malhas foi mantido a 300µm para o diâmetro de cada fio. O teste *peel load* foi aplicado ao modelo para investigar as tensões geradas nas diferentes camadas.

**RESULTADOS:**

quando comparadas às bases de braquetes de malha simples, as bases de braquetes de malha dupla geraram menos tensão no esmalte dentário. Não foram detectadas diferenças entre as tensões geradas na superfície dos braquetes com bases de malha simples e dupla. Na malha de fios impregnados (MFI), houve um aumento na tensão com o aumento do diâmetro dos fios que compõem a malha.

**CONCLUSÃO:**

os resultados revelam que as modificações no desenho do braquete podem aumentar a colagem e, ao mesmo tempo, minimizar os danos causados no esmalte durante o processo de descolagem. Esses fatos podem ser utilizados no desenvolvimento de desenhos de braquetes inovadores, destinados à utilização clínica.

## INTRODUCTION

The key to successful malocclusion correction is the application of sustained force. Force is applied to teeth via brackets, thus, brackets play a major role in the system of correction of malocclusion. 

Bonding has been a boon granted to the branch of Orthodontics since its introduction by Buonocore.^1^ It has solved the major problem of attaching brackets to teeth. Newman was the first to directly bond brackets to the enamel surface;^2^^,^ 3 however, problems were persistent. As more and more adults started enjoying the benefits of Orthodontics, the problem of visibility of metal brackets surfaced.

An obvious choice to overcome this was the use of esthetic brackets (ceramic, plastic, etc.) and lingual Orthodontics, both of which had their own set of disadvantages and advantages. Ceramic brackets, having a chemical bond with teeth, posed the problem of enamel damage during debonding as well as increased brittleness leading to wing fracture.^4^^-^ 8 In addition, there is the issue of frictional resistance and iatrogenic enamel damage.^9^ Lingual Orthodontics can be performed in selected cases. Overtime, most disadvantages related to ceramic brackets were quite effectively addressed. Nevertheless, the technique never met the gold standard of metal brackets, as it clearly lacked their ductility. In order to overcome the issue of enamel damage caused by ceramic brackets debonding, many adhesive material^10^ and debonding techniques^11^ (laser operate debonding) have surfaced. Nevertheless, that again is an addition to the inventory, which can be an economical burden to orthodontists as well as patients. Thus, metal brackets still dominate the scene with their intact gold standard. With a view to rendering metal brackets more patient-friendly, their bulk was significantly reduced and mini brackets made their way into the field.

Logically speaking, reducing the bulk resulted in decreased surface area for bracket bonding, which significantly affects bond strength.^12^ This has paved the way for researchers to study different bracket modifications so as to improve bond strength. Gradual evolution in the context of bracket material and mesh design is an inevitable change. Considering that the ideal bracket requirement does not change much, it should have the adequate bond strength to withstand the forces of the wire, in addition to causing minimal damage to the enamel while debonding. Meanwhile, it should not be bulky enough so as to compromise patient's esthetics.^13^ Production of such a bracket is the requirement of the day.

Studying such complex designs *in vivo* is a time-consuming and tedious work. Virtual models are ideal to deal with complex set ups within time constraints and without much economic burden. To date, the most popular virtual modelling system prevalent in the field of Orthodontics is the finite element method (FEM).^14^^-^ 18 FEM analyzes the stress distribution factor of different components, thus enabling researchers to understand the practicality of using certain models.

Studying stress distribution in different layers of bracket bonding systems, i.e bracket-cement-tooth system, may give us the insight into the potential possibility of producing an ideal bracket system. In this context, many studies explored the possibilities of bracket modification, including the double-mesh bracket base.^18^^-^ 24 Double-mesh bracket studies have divided the double-mesh layers as coarse and fine mesh. These studies report that in the superficial layer of the double-mesh bracket, stress was reduced.^18^ This fact did not put much light on the stress produced on the other layers of the bracket-cement-tooth interface. Presently, there is a need for a technological revolution aiming at achieving favorable clinical outcomes in the field of bracket mesh base design. The present article enjoys the benefits of the finite element method to construct a computerized three-dimensional virtual model of bracket-cement-tooth interface with a view to assessing and analyzing stress distribution produced by modifying the bracket base geometry in single-mesh bracket base, and to compare it with the double-mesh bracket base design using peel load, all of which to bring about the favorable bracket mesh base design.

## MATERIAL AND METHODS

The geometric image of a maxillary first premolar was determined by taking 0.5-mm longitudinal sections of a representative tooth by means of computer tomography (General Electronics, USA). These sections were then transferred to AutoCAD software (Autodesk Inc., USA) to get the geometric model of the maxillary first premolar. The model generated was transferred to a finite element package in IGES (initial graphics exchange specification) format. IGES files are neutral files that can support almost all CAD software and are also amenable for analysis.

Using digital measurements of these sections, the three-dimensional coordinates of the tooth were recorded and a finite element mesh was generated using a commercial mesh generating programmer (ANSYS 7.0). Only the area of the tooth required for bracket placement was generated and secured by appropriate boundary conditions. This helped to reduce the size of the overall model.

**Figure 1 -:**
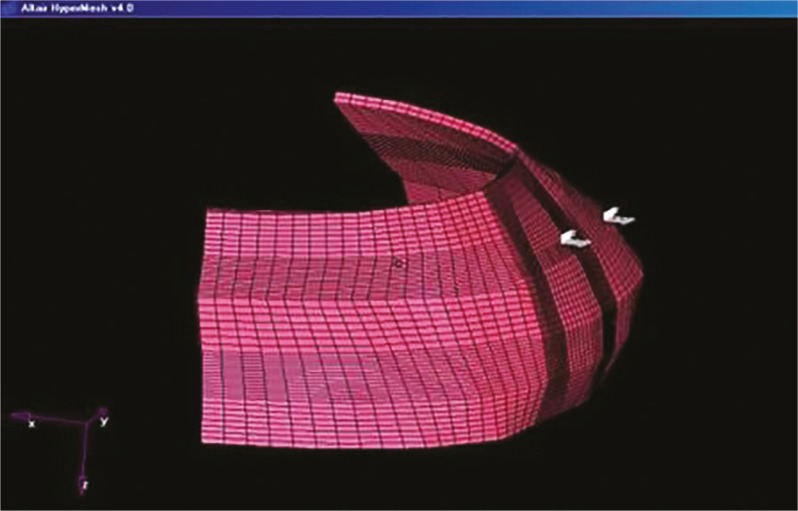
Finite element model of enamel.

**Figure 2 -:**
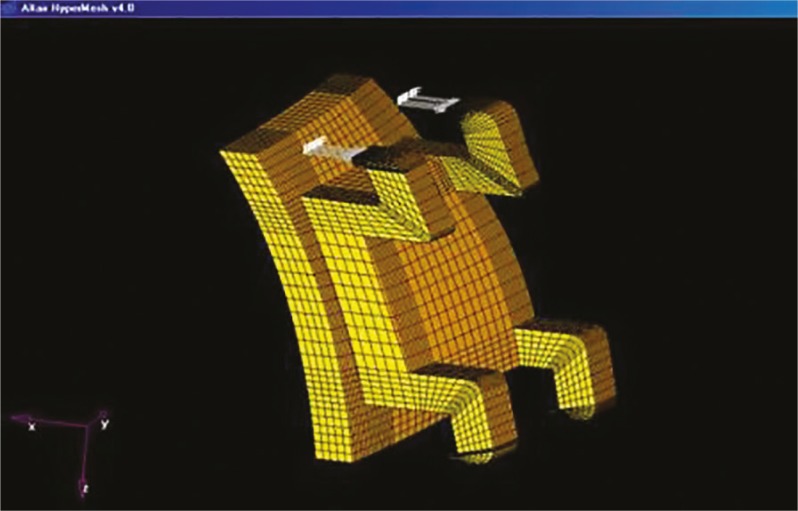
Finite element model of bracket.

**Figure 3 -:**
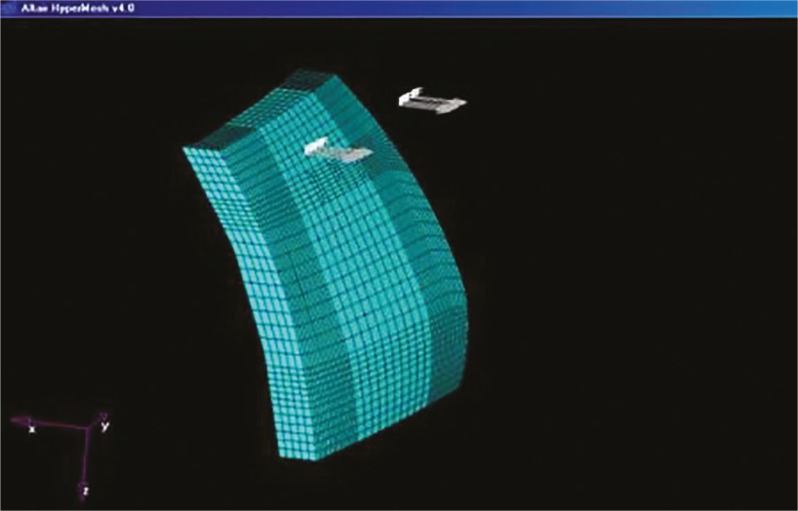
Finite element model of IWM.

A maxillary first premolar bracket (MBT bracket system, Ortho Organizer) was modeled using the geometric measurements obtained by the digital vernier caliper. Apart from the tooth and bracket, an impregnated wire mesh (IWM) layer was constructed using previous data from the literature (Figs 1, 2, 3).^18^^,^ 24 ^,^ 25 IWM is a layer where cement and metal mesh are joined or intermingled. All layers of the tooth-IWM-bracket system were kept linear, elastic, isotropic and homogeneous. Theory of composite material was applied to generate the properties of IWM layer as per the recommendation of earlier studies of similar nature (Table 1).^18^^,^ 24 ^,^ 25

**Table 1 -:** Material properties employed

Material	Young’s modulus (MPa)	Poisson’s ratio
Enamel	46.890	0.30
Cement	11.721	0.21
Stainless steel	210.00	0.30

**Figure 4 -:**
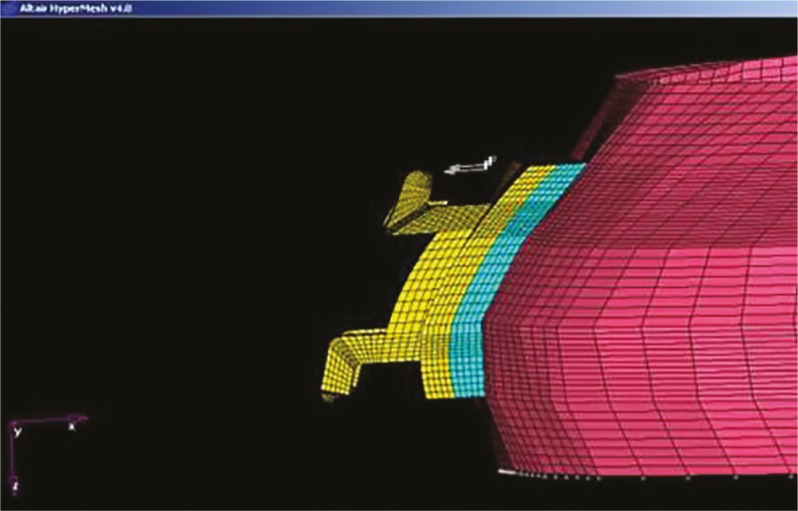
Finite element model of the tooth-cement-bracket continuum.

The material parameters used in the computations are similar to those used in previous studies.^24^^,^ 25 However, Poisson's ratio for IWM for each modification was calculated separately for single and double-mesh bracket base models, as depicted in Tables 2 and 3, respectively. The complete three-dimensional finite element model of the bracket-cement-tooth system consisted of 40,536 bonds and 49,201 finite elements (Fig 4). The mesh base is the crisscross of stainless steel wire with a gap between the wire for cement retention. The geometry of the mesh base was altered by increasing the mesh wire diameter sequentially from 100 µm to 400 µm consecutively, while spacing was kept constant at 300 µm.

**Table 2 -:** Material properties of IWM layer in single-mesh bracket base for different diameters and spacing.

Diameter (µm)	Spacing (µm)	Length (µm)	Width (µm)	Area (µm^2^)	Long. deflection	Lat. deflection	E	Long. strain	Lat. strain	Poisson’s ratio
100	300	200	400	160000	1.18E-15	3.10E-16	1.059E+08	5.900E-18	7.750E-19	0.131
200	300	400	500	250000	1.32E-15	3.46E-16	1.212E+08	3.300E-18	6.920E-19	0.210
300	300	600	600	360000	1.22E-15	3.23E-16	1.366E+08	2.033E-18	5.383E-19	0.265
400	300	800	700	490000	1.07E-15	2.84E-16	1.526E+08	1.338E-18	4.057E-19	0.303

**Table 3 -:** Material properties of IWM layer in double-mesh bracket base for different diameters and spacing.

Diameter (µm)	Spacing (µm)	Length (µm)	Width (µm)	Area (µm^2^)	Long. deflection	Lat. deflection	E	Long. strain	Lat. strain	Poisson’s ratio
100	300	400	400	160000	2.04E-15	3.86E-16	1.225E+08	5.100E-18	9.650E-19	0.189
200	300	800	500	250000	2.09E-15	3.59E-16	1.531E+08	2.613E-18	7.180E-19	0.275
300	300	1200	600	360000	1.86E-15	3.21E-16	1.792E+08	1.550E-18	5.350E-19	0.345
400	300	1600	700	490000	1.61E-15	2.80E-16	2.028E+08	1.006E-18	4.000E-19	0.398

The guidelines from a previous study were taken into consideration to prepare the double-mesh base geometry.^18^ Each layer was homogenized separately before introducing them into the overall FE model.

To assess the stress generated by altering the geometry of the bracket mesh base, peel load of 1 N was used (Fig 4). The obtained results were tabulated and subjected to percentile calculation for comparison of single and double-mesh bracket bases for different layers of tooth-cement-bracket continuum.

## RESULTS

**Figure 5 -:**
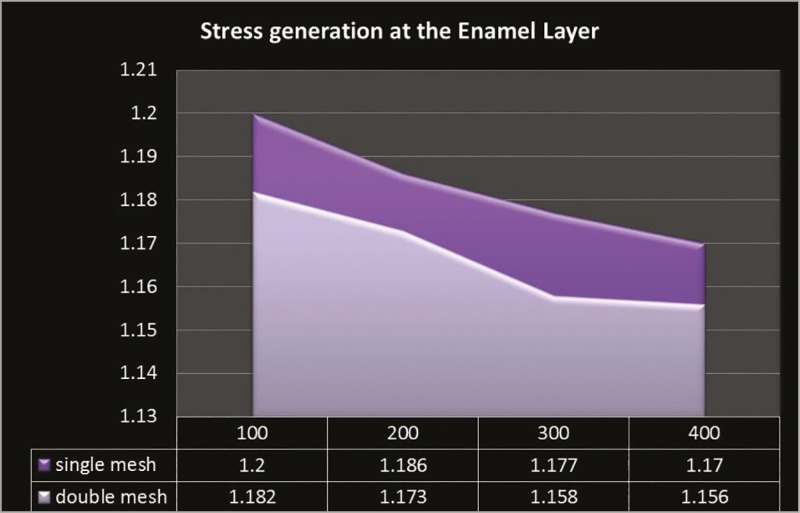
Comparison of stress generated at the enamel layer for singleand double-mesh bracket bases.

The results are represented in the form of charts. Figure 5 represents the difference in the stresses generated at the enamel layer for single and double-mesh bracket bases. Stress was higher on enamel as the wire diameter decreased. The single mesh produced more stress on the enamel than the double-mesh bracket base.

**Figure 6 -:**
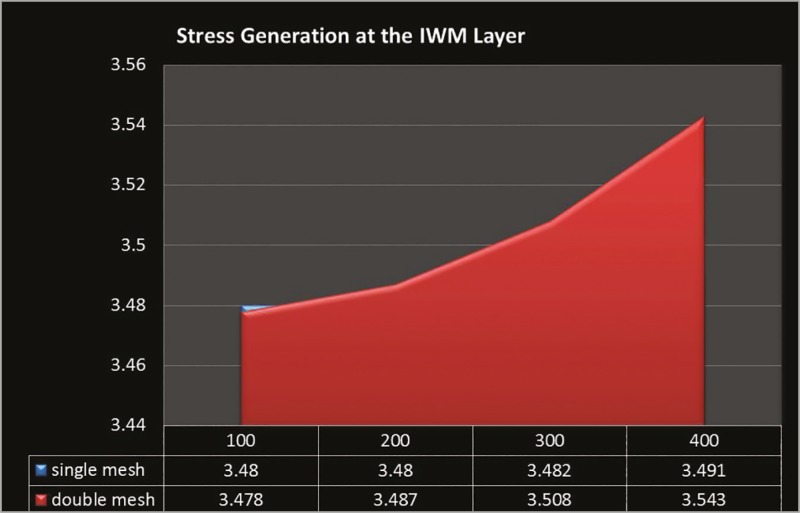
Comparison of stress generated at the IWM layer for single and double-mesh bracket bases.

The range of stresses for the IWM layer in single and double-mesh bracket bases is depicted in Figure 6. Stresses nearly remained the same for single and double-mesh bracket bases, but were high on IWM when wire diameter increased.

**Figure 7 -:**
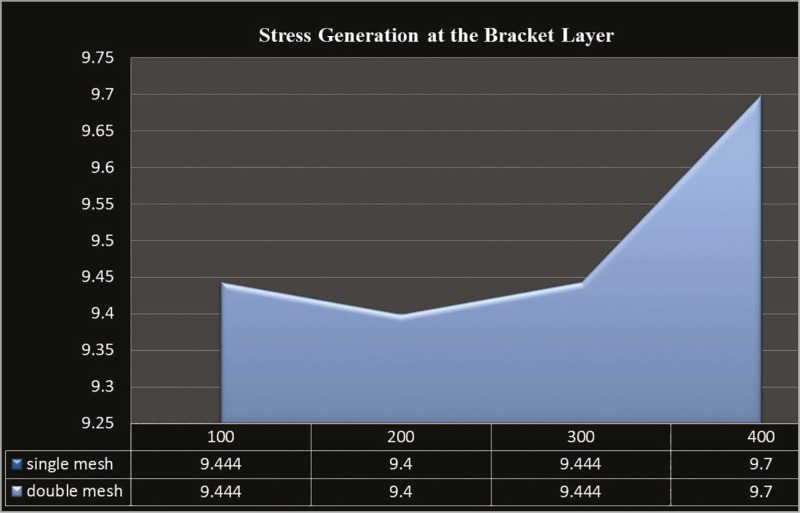
Comparison of stress generated at the bracket layer for singleand double-mesh bracket bases.

For the bracket layer of the single and double-mesh base model, stress remained constant, as presented in Figure 7. Stress ranged from 9.4 to 9.7 MPa and remained the same for both single and double-mesh bracket systems.

## DISCUSSION

The study used a three-dimensional finite element model of the tooth-bracket-cement system to assess the stress generated by altering the mesh base design. A peel force of 1 N was applied and the stresses generated were registered.

**Figure 8 -:**
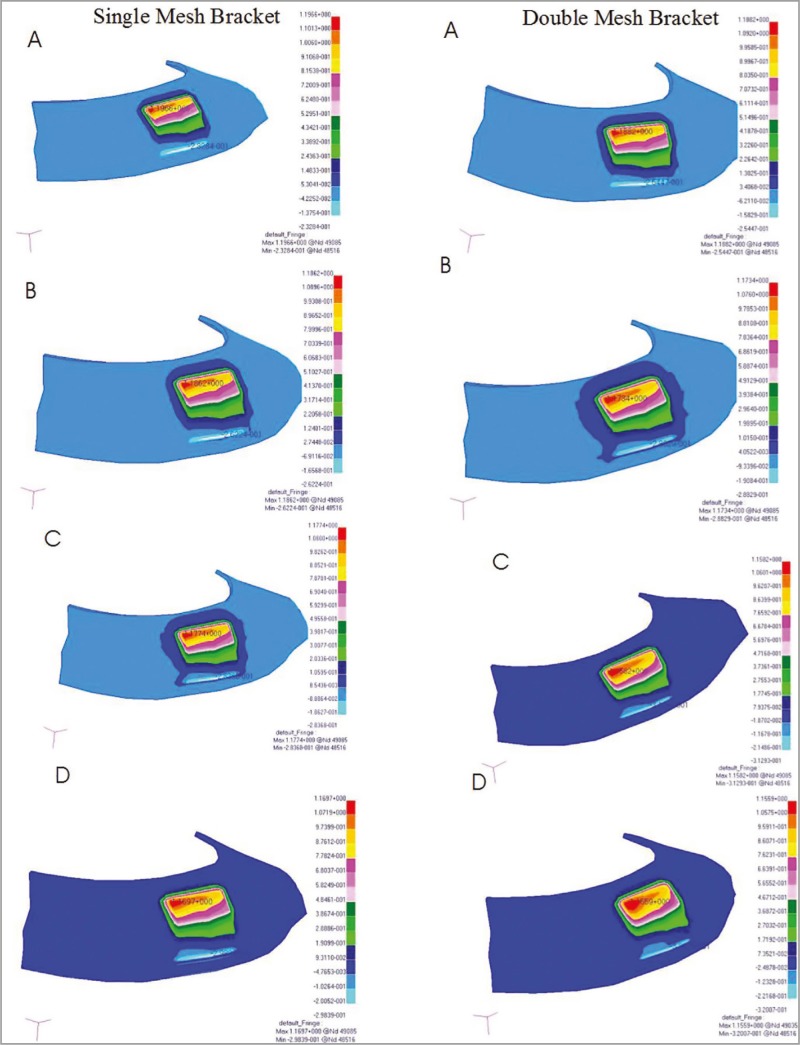
Stress on enamel at different wire diameters of the mesh for single and double-mesh bracket bases.

The stress generated in the enamel layer of the single-mesh bracket base model decreased progressively as the diameter of the mesh wire increased (Fig 8). As the wire diameter of the mesh base increased, the surface area also increased, thus, inuring the distribution of force evenly over the large surface. This is probably the reason behind the decrease in stress on enamel, as the wire diameter of the bracket mesh base increases.

A similar phenomenon was noticed in the double-mesh bracket base at the enamel layer (Fig 8). However, when single and double-mesh bracket bases were compared, the stress in the double-mesh bracket base at the enamel remained low in comparison to the single-mesh bracket modification. This assures less damage to the enamel layer while orthodontic bracket debonding procedure is carried out. Double-mesh bracket design has greater surface area in comparison to the single-mesh bracket base, thus, stress distribution on the mesh is generous, which ensures less stress concentration on the enamel.

Nevertheless, a previous study checking the efficiency of different bracket designs showed that double-mesh bracket produced greater bond strength in comparison to other bracket designs.^26^

Of all the different layers of the FEM model applied to the bracket-cement-tooth continuum, the stress generated at the bracket remained high for both single and double-mesh bracket bases. The point of force application is on the bracket and, owing to this factor, the stress generated at the bracket was greater.

In case of an IWM layer, stress increased progressively with the increase in wire diameter for both models. As wire diameter increased, the part of the impregnated wire mesh constituted by the cement decreased and there was a smaller area of cement impregnating the wire mesh, which can take up the stress. This criterion led to the increase in stress at the IWM layer as the wire mesh diameter increased.

Further, previous researchers have shown that the success of bracket base design in increasing bonding strength is not only dependent on the bracket base, but also on the type of bonding agent selected. Additionally, certain brackets performed well with a particular brand of bonding agent.^27^

When one has the bird view of the stress generated in both models, it is evident that maximum stresses were noticed at the bracket, followed by the IWM layer of the tooth-cement-bracket continuum. This indicates the possible fracture site of the continuum when the debonding procedure is performed. Nevertheless, the above point is advantageous for the orthodontist, as one can safeguard the enamel wear and tear, which ultimately is the concern of every orthodontist.

As the wire diameter increased, the possible retentive unit area for the cement decreased and the load was taken up by the increased surface area of the wire, which in turn produced less impact on the enamel. With all due respect to the above finding, one has to ponder around the fact that the profile of the bracket might increase significantly with double-mesh design.

The results of the present study indicate that altering the mesh geometry affects the bonding strength of the bracket. Both contrasting and accordance evidence was found in earlier studies of similar nature.^21^^,^ 23 Nevertheless, for better bonding, with smaller chances of enamel damage during the debonding procedure, double-mesh bracket base can be an ideal choice.

A previous study reports that single and double-mesh bracket bases had comparable bonding strength and bracket failure modes.^19^ This study is quite contrasting to the findings of the present study, as there existed a difference in stress noted in different layers of the tooth-cement-bracket system.

Other than wire diameter and wire spacing, the researchers have identified a number of variables in the bracket mesh which might exert some influence on the bonding strength of the bracket, namely: weld spots, weld spurs, location of weld spots and air entrapment.^20^ While the present study mainly emphasized the difference in the behavior of single and double-mesh bracket bases, the above mentioned variables should be taken into consideration and a study of more extensive nature should be conducted.

## CONCLUSION

Modifying the bracket mesh base by varying the diameter of the wire mesh significantly influences the amount of stress generated in the bracket-cement-tooth continuum.

The double-mesh bracket base can be an answer for the potential reduction of enamel wear and tear during debonding.

Further in-depth investigations are needed on other bracket base mesh designs and related variables influencing them, as there are relatively few studies in this regard. This study can be used as reference for future investigation.

In today's world of inventory abundance, the orthodontist should be well equipped with evidence-based material to be for individual cases. The present article tried to address past unsolved issues of bonding strength and found the solution which will guide the clinician to choose the best bracket mesh base for efficient bonding with least enamel damage possible during debonding processes.
